# Fecal bacterial microbiota of Canadian commercial mink (*Neovison vison*): Yearly, life stage, and seasonal comparisons

**DOI:** 10.1371/journal.pone.0207111

**Published:** 2018-11-12

**Authors:** Nicole R. Compo, Diego E. Gomez, Brian Tapscott, J. Scott Weese, Patricia V. Turner

**Affiliations:** 1 Department of Pathobiology, University of Guelph, Guelph, Ontario, Canada; 2 Ontario Ministry of Agriculture, Food, and Rural Affairs, Elora, Ontario, Canada; University of Porto, PORTUGAL

## Abstract

The gastrointestinal microbiome is known to play a critical role in animal health but has been relatively poorly characterized in commercial mink, an obligate carnivore. Whether the microbiota can be manipulated in mink to improve pelt quality, health, and well-being is unknown. The objectives of this study were to characterize the fecal microbiota of commercial mink, and to evaluate potential changes due to year (2014 vs 2015), life stage (adult female vs weaned kit), season (summer vs winter), and between Canadian farms. Pooled fecal samples were collected from adult females and weaned kits in the summers of 2014 (n = 173) and 2015 (n = 168), and from females in the winter of 2016 (n = 39), a time when females undergo marked calorie restriction, from 49 mink farms in Ontario. Bacterial DNA was extracted and the V4 region of the 16S rRNA gene was amplified. Approximately 22 million sequences were identified following quality control filtering. A total of 31 bacterial phyla were identified; however, only 3 comprised >1% of the total sequences identified, with Firmicutes and Proteobacteria together comprising 95% of the total sequences. Comparisons were made by life stage, season and year; no differences were found in the relative abundance of any taxa between samples collected from adult females and weaned kits from the same year and the greatest number of differences at each taxonomic level were noted between 2014 and 2015. Significantly more operational taxonomic units (OTUs) were found in 2014 than 2015 or 2016 *(p*<0.05) and samples from 2014 were more even, but less diverse than in 2015 (*p* = 0.002 and 0.001, respectively). There were significant differences in community population and structure by year and season (all *p*-values <0.001). The predominant phyla and genera at the farm level were similar from year to year. Together, these indicate that mink environment, season, and time are important factors in the stability of gastrointestinal microbiota, once mink reach maturity.

## Introduction

The intestinal microbiome represents the collective interacting genomes and symbiotic microorganisms in the gastrointestinal tract [1]. In recent years, the integral role of the gut microbiota in disease, health, and development has been well established in a variety of species, including humans, mice, dogs, and pigs [2–6]. To date, most studies have focused on herbivores and omnivores and few studies have been conducted in carnivores. Moreover, with the exception of a few studies in domestic cats, prior studies conducted in carnivores have been based on a limited number of samples (i.e. ten or fewer) [7–9].

Mink (*Neovison vison*) are an important commercial farmed livestock group in Canada, and have a short and simple gastrointestinal tract, resulting in a relatively fast total food transit time [10,11]. The significance of this relates to the amount of time bacteria have to assimilate nutrients, as a faster transit time reduces bacterial production of short chain fatty acids and digestion of amino acids. In humans, a faster gastrointestinal transit time leads to a significantly reduced bacterial mass, but whether this has an impact on the overall composition of the fecal microbiota is unknown [12].

In a small sample of ferrets (a closely related mustelid to mink) only one organism, *Clostridium acetobutylicum* (phylum Firmicutes), was cultured from >50% of fecal samples, suggesting a reduced bacterial microbiota compared with other mammals [13]. Other species identified included other members of Firmicutes, as well as members of Actinobacteria and Proteobacteria, findings similar to other carnivores, regardless of the use of bacterial culture or culture-independent techniques [13]. Using bacterial culture on fecal swabs from farmed mink in Denmark, Vulfson *et al* isolated *Escherichia coli*, enterococci, and lactic acid bacteria from 50%, 90%, and 90% of samples, respectively [14]. However, culture-dependent techniques are generally unable to provide a detailed or even necessarily accurate reflection of the complex fecal microbiota. This is reflected in the discrepancy identified by Bahl *et al* between the organisms identified from the intestinal mucosa of mink by microbiologic culture and those identified by 16S rRNA gene sequencing [15]. In their study, Clostridia were underrepresented by culture, but made up a large proportion of the sequences identified by next generation sequencing [15]. This provides evidence that many species of bacteria have likely been underrepresented in culture-based studies and, thus, their importance to the gastrointestinal microbiota is undetermined.

It is unclear if the gut microbiota of carnivores is as extensive, functional or influential on the nutritional status and overall productivity of the host as in non-carnivores. In one study, the large intestinal microbiota of the cat was demonstrated to be necessary for optimal utilization of nutrients, and was affected by diet [16]. However, this contrasts with results in gnotobiotic ferrets, which had minimal morphologic or metabolic changes with a selected, rather than conventional, gut microbiota [17]. Moreover, mink have substantially fewer colonic bacteria (2–4 orders of magnitude) than most other mammals, likely attributable to their fast gut transit time [18]. Similarly, studies to date have not explored the changes of the fecal microbiota, if any, with diet in carnivores other than cats. Food sources for the commercial mink industry are heavily reliant on the availability of protein sources, which can change substantially from year to year or even season to season, and it is unclear whether the diet changes impact the gut microbiota of these animals. As such, there is a need to more fully understand the role of the gut microbiota in the carnivore.

Knowledge of the gastrointestinal microbiota of other farmed species has been used to optimize various production parameters and improve overall animal welfare. Chicks have been shown to be resistant to *Brachyspira* sp. pathology when orally inoculated with *Lactobacillus reuteri* [19]. Both generic and chick-specific probiotics increased the overall productivity of broiler chickens, with the chicken-specific probiotic having a more enhanced effect [20]. Similarly, *Pediococcus acidilactici*-supplemented piglets demonstrated higher body weights, post-weaning daily weight gain, and number of proliferating enterocytes compared to untreated controls [21]. Investigations into how the microbiota of farmed mink could be optimized to improve health and production parameters have yet to be determined.

The objectives of this study were to characterize the fecal microbiota of commercial mink raised on Canadian farms, and with the resulting data, to compare the microbiota of adult females and weaned kits in summer, adult females between summer and winter, and to determine if the fecal microbiota is similar at the farm-level from year-to-year (during summer). Based on results from other mammalian species, we hypothesized that the predominant phyla in mink feces would be Firmicutes and Proteobacteria and that adult females and weaned kits would have similar fecal microbiota, particularly within a farm. Additionally, we expected that because of differences in diet, the impact of summer heat stress, and altered nutrition of overwintered females kept for breeding that there would be significant differences seen between summer and winter results.

## Materials and methods

### Farm recruitment and sample collection

Ontario mink producers were contacted through the Ontario Fur Breeders Association (OFBA) and the Ontario Ministry of Agriculture, Food, and Rural Affairs (OMAFRA) for potential study enrollment. Enrollment was voluntary and at the discretion of the farm owner. A total of 43 farms were initially enrolled, representing >93% of Ontario mink farms in 2014. Over the three-year study period, several farms pelted out, only participated in one year of the study or were new farms, such that a total of 49 different farms participated: 43 in 2014, 46 in 2015, and 39 in 2016. Because fecal samples were collected from under the cages of mink by the farm owners for this study with no manipulation of animals, no institutional animal ethical review was required.

Sample collection was performed by a government-industry technical representative (BT) in collaboration with farm owners and occurred between July and October of 2014 and 2015 and from January to February of 2016. Samples from a single farm were collected on the same day, over 12, 13, and 10 days (non-consecutively) in 2014, 2015, and 2016, respectively. Fresh fecal samples were collected from beneath three cages of females, containing 1–2 females per cage, and pooled, such that each sample represented 3–6 females. The process was repeated for weaned kit fecal samples (n = 3–4 kits/cage). Two pooled samples from females and two pooled samples from weaned kits were collected from each farm in 2014 and 2015, with the exception of five farms; three farms in 2014 had only two pooled samples collected (1 female, 1 weaned kit) and two farms in 2015 had only one pooled female sample collected. For the winter 2016 sample collection, one pooled female sample was collected from each farm. The total number of samples collected each year is summarized in Table 1. Samples were collected and delivered to the University of Guelph, where they were coded to preserve anonymity and stored at -80°C pending processing. Later, samples were thawed at room temperature and 0.2 g of feces was aliquoted and refrozen at -80°C pending DNA extraction.

**Table 1:** Number of pooled fecal samples collected from adult females and weaned kits collected from commercial mink farms in Ontario.

Year & season (number of farms)	Adult females (n = 212)	Weaned kits (n = 175)
2014 summer (43)	83	83
2015 summer (46)	90	92
2016 winter (39)	39	not collected

### DNA extraction, amplification, and sequencing of bacterial 16S rRNA gene

DNA was extracted using the E.Z.N.A. Stool DNA Kit (Omega Bio-Tek, Inc, Doraville, Georgia, USA) according to the manufacturer’s instructions using 0.2 g of feces. A negative control for DNA extraction was not included. Following extraction, spectrophotometry (NanoDrop, Roche, Mississauga, ON, Canada) was used to assess the quantity of nucleic acids present.

The V4 region of the 16S rRNA gene was amplified using a published PCR protocol [22] and the following primers: forward S-D-Bact-0564-a-S-15 (5′-AYTGGGYDTAAAGNG-3′) and reverse S-D-Bact-0785-b-A-18 (5′- TACNVGGGTATCTAATCC-3′). Both primers were designed with regions that overlap with the Illumina sequencing primers (Forward: TCGTCGGCAGCGTCAGATGTGTATAAGAGACAG, Reverse: GTCTCGTGGGCTCGGAGATGTGTATAAGAGACAG), to allow for annealing to the Illumina universal index sequencing adaptors, plus the 8 bp identifier indices (Forward: AATGATACGG CGACCACCGAGATCTACAC-index-TCGTCGGCAGCGTC, Reverse: CAAGCAGAAGACGGCATACGAGAT- index-GTCTCGTGGGCTCGG). For each sample (and one negative control of sterile water on each day PCR was conducted), a reaction mixture was prepared using 12.5 μl KAPA Ready Mix, 9.0 μl sterile water, 0.5 μl each of the forward and reverse primers (10 pM/μl), and 2.5 μl of the extracted DNA (5 ng/μl) (or sterile water) and the PCR parameters were as follows: 1) 3 min at 94°C for denaturation, 2) 45 sec at 94°C for denaturation, 3) 60 sec at 53°C for denaturation, 4) 1.5 min at 72°C for elongation, and 5) 10 min at 72°C. Steps 2–4 were repeated for a total of 27 cycles. To ensure that bands of appropriate length (~254 bp) were present following the first PCR, electrophoresis of the PCR product was completed in a 2% agarose gel. PCR products were stored at 4°C until purification.

Following purification of PCR products with Agencourt AMPure XP (Beckman Coulter Inc., Mississauga, ON, Canada), a second PCR was completed using 2.5 μl of the purified product or sterile water (negative control), 12.5 μl KAPA Ready Mix, 9.0 μl sterile water, and 1.0 μl of the Illumina Forward and Reverse Index Primers (I501-I508 or S513, S15-S18, S20-S22 and I701-712 or N716, N718-N729, respectively). The PCR parameters were as follows: 1) 3 min at 94°C, 2) 45 sec at 94°C, 3) 60 sec at 50°C, 4) 1.5 min at 72°C, and 5) 10 min at 72°C. Steps 2–4 were repeated for a total of 8 cycles. The second PCR product was purified as above, with 40 μl of AMPure XP and 35 μl 10mM Tris pH 8.5 Buffer. The final purified products were evaluated for bands of appropriate length using 2% gel electrophoresis. When appropriate bands were not observed, the first amplicon was quantified using spectrophotometry and reamplified following appropriate adjustments to the volumes of KAPA Read Mix, DNA product, or sterile water to correct for this.

Samples were normalized to a final concentration of 12 nM. The library pool was submitted to the University of Guelph’s Advanced Analysis Centre and sequenced with an Illumina MiSeq (Illumina RTA v1.17.28, San Diego, California, USA) for 250 cycles from each end.

### Sequence processing and statistical analyses

Microbiota analyses were completed using mothur software (v 1.38; https://www.mothur.org) [23]. Paired end reads were aligned and sequences that contained ambiguous bases, were longer than 275 bp, or that contained homopolymer runs > 8 bp were removed. Paired-end reads were aligned to the SILVA 16S rRNA reference database [24] and any that were misaligned with the target region were removed. Chimeras and non-bacterial sequences (i.e. chloroplasts, mitochondria, Archaea, and Eukaryotes) were removed. Sequences were identified using the RDP classifier [25] and binned into phylotypes using a closed OTU-picking approach.

The relative abundance of the predominant phyla, classes, orders, families, and genera were calculated and stacked column graphs were created. A Wilcoxon rank-sum test was used to compare the relative abundance of different taxa between seasons (summer vs winter, adult females 2015 and 2016 only, n = 117; hereafter referred to as “season”) and life stages (adult females vs weaned kits, 2014 and 2015 only, n = 332; hereafter referred to as “life stage”) by using JMP 12 Response Screening Platform (SAS Institute Inc., Cary, NC). A Wilcoxon rank-sum test for multiple comparisons was used to test by life stage and year (adult females 2014 vs 2015, n = 164; weaned kits 2014 vs 2015, n = 168) and by farm between years. Only those farms for which all 8 samples were available (i.e. 2 adult females and 2 adult kits, for both 2014 and 2015) were used for farm-level comparisons (n = 8 for each farm x 30 farms). Multiple comparison adjustments were conducted using the Benjamini and Hochberg’s False Discovery Rate (FDR), with an adjusted *p*< 0.05 accepted as statistically significant.

Subsampling of 11,000 sequences, chosen based on the minimum number of sequences present in any sample following quality control filtering, was performed to normalize sequence numbers. Sampling coverage was assessed by Good’s Coverage. Measures of alpha diversity, inverse Simpson’s, Chao1, and Shannon’s evenness indices, were used to calculate diversity, richness, and evenness, respectively. Beta diversity was measured using the Jaccard and Yue and Clayton indices, measures of community population, and population and structure, respectively, and used to create dendrograms using FigTree (v.1.4.3; http://tree.bio.ed.ac.uk). Analysis of molecular variance (AMOVA) and parsimony tests were used to compare year, life stage, and season on both the Jaccard and Yue and Clayton indices. Visual similarities and clustering of each of these groups was plotted with principal coordinate analyses (PCoA). Within mothur, linear discriminant analysis effect size (LEfSe) [26] was performed to identify bacterial taxa that were differentially abundant between groups.

## Results

### Mink fecal microbiota: Overall assessment

A total of 366 mink fecal samples were included in the final analyses; 198 from adult females and 168 from weaned kits (Table 2). Twenty-one samples were not included due to poor DNA yield after extraction or a repeated inability to obtain adequate sequence numbers. Following quality control filtering, a total of 22,238,782 sequences were yielded, with a range of 9,692 to 205,174 (median 57,953) sequences per sample. A subsample of 11,000 reads/sample was used to normalize sequence numbers across samples and was considered adequate, as evidenced by greater than 99% coverage for all samples and plateau of rarefaction curves (raw data accessible at Scholars Portal Dataverse, [27]).

**Table 2:** Number of samples used in the final analysis of the fecal microbiota of commercial mink.

Year & season (number of farms)	Year & season	Adult females (n = 198)	Weaned kits (n = 168)
2014 summer (43)	2014 (summer)	81	82
2015 summer (46)	2015 (summer)	83	86
2016 winter (39)	2016 (winter)	34	not collected

**Fig 1:**
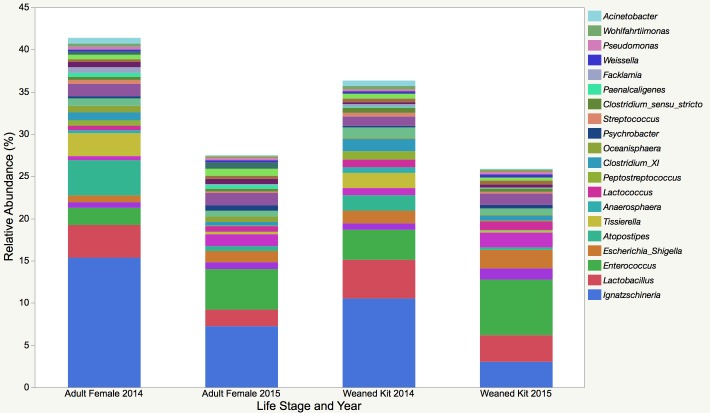
Relative abundance of the 20 most predominant bacterial genera present in the feces of commercial mink (n = 366). Samples were obtained from 43, 46, and 39 farms in 2014, 2015, and 2016, respectively.

A total of 31 bacterial phyla were identified; however, only 3 consisted of >1% of the total sequences identified, with Firmicutes and Proteobacteria together comprising 95% of the total sequences. Greater than 1300 genera were identified; the 20 overall most predominant genera are depicted in Fig 1 by year and life stage and by farm in the supplementary material (S1 Fig). The most abundant genera include *Ignatzschineria*, *Lactobacillus*, and *Enteroccoccus*. with their corresponding families, orders, and classes also predominating (Table 3).

**Table 3:** Median relative abundance of predominant taxonomic classifications of bacteria from the feces of commercial mink (n = 366). Samples were obtained from 49 farms in the summers of 2014 and 2015 and winter 2016.

Classification level	Taxon	Relative abundance (%)
Phylum (>1%, among 31 identified)	Firmicutes	60.5
	Proteobacteria	34.4
	Bacteroidetes	1.7
Class (>1%, among 83 identified)	Bacilli	43.7
	Gammaproteobacteria	30.9
	Clostridia	15.2
	Betaproteobacteria	1.7
	Alphaproteobacteria	1.5
	Flavobacteria	1.4
	Erysipelotrichia	1.2
Order (>1%, among 148 identified)	Lactobacillales	33.7
	Xanthomonadales	15.2
	Clostridiales	15.1
	Bacillales	9.3
	Enterobacteriales	6.4
	Pseudomonadales	5.4
	Aeromonadales	2.0
	Burkholderiales	1.6
	Flavobacteriales	1.4
	Erysipelotrichales	1.2
Family (>2%, among 336 identified)	Xanthomonadaceae	15.2
	Lactobacillaceae	12.8
	Enterococcaceae	7.1
	Enterobacteriaceae	6.4
	Peptostreptococcaceae	4.8
	Carnobacteriaceae	4.7
	Planococcaceae	4.3
	Clostridiales_Incertae_Sedis_XI	4.0
	Streptococcaceae	3.6
	Moraxellaceae	2.9
	Pseudomonadaceae	2.5
	Staphylococcaceae	2.4
	Incertae_Sedis_XI	2.3
	Clostridiaceae_1	2.2
	Aerococcaceae	2.2
	Aeromonadaceae	2.0
Genus (>2%, among 1,255 identified)	*Ignatzschineria*	14.0
	*Lactobacillus*	8.0
	*Enterococcus*	5.4
	*Escherichia_Shigella*	4.3
	*Atopotipes*	3.6
	*Tissierella*	2.8
	*Anaerosphaera*	2.1
	*Lactococcus*	2.1
	*Peptostreptococcus*	2.1
	*Clostridium*_cluster XI	2.0

### Mink fecal microbiota: 2014 vs 2015

**Fig 2:**
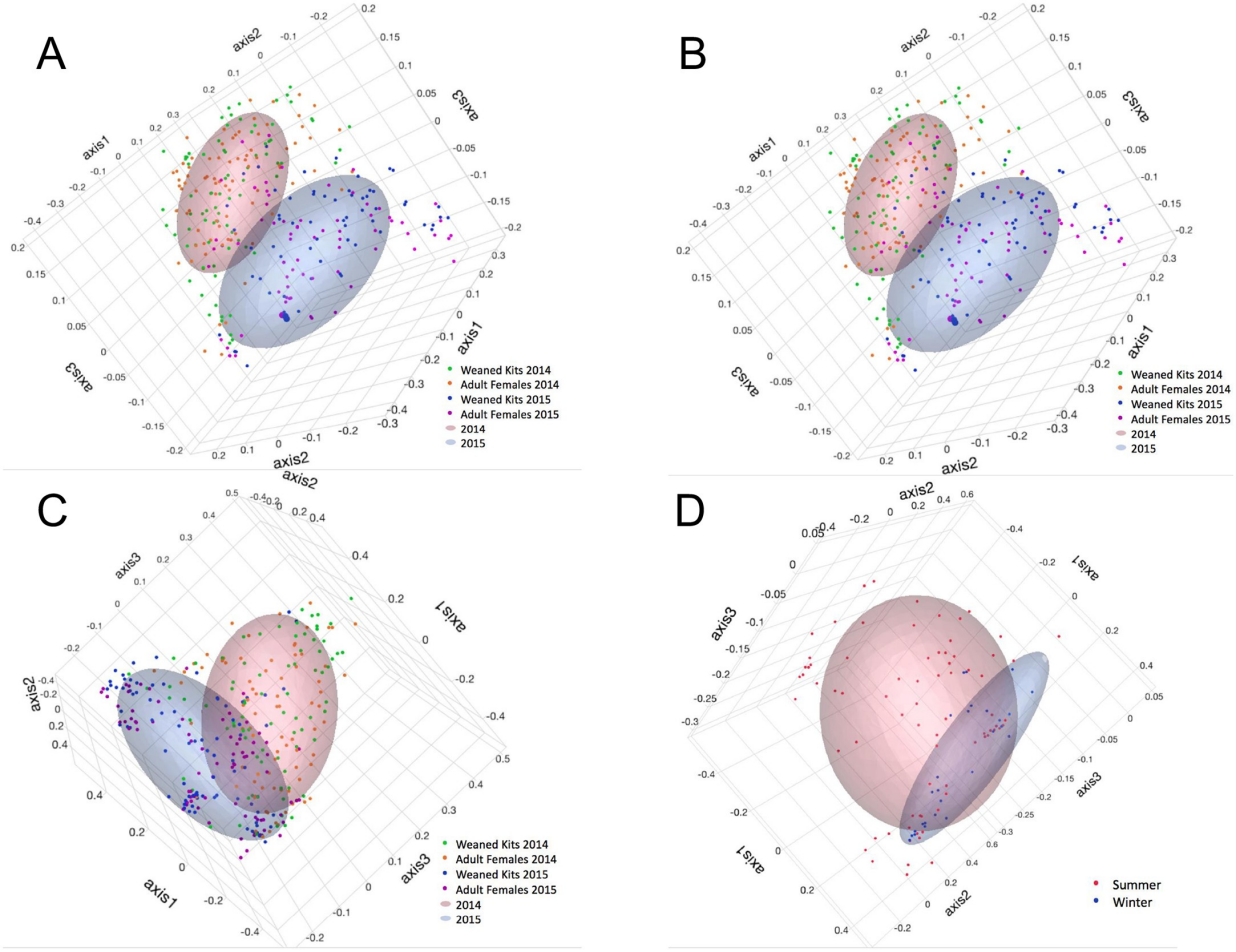
Principal coordinate analysis (PCoA) of community population based on the Jaccard Index (a and b) and community structure based on the Yue-Clayton Index (c and d) of the fecal microbiota of commercial mink, by year (n = 332) (a and c) and season (n = 117) (b and d). Samples were obtained from 43, 46, and 39 farms in 2014, 2015, and 2016, respectively.

The composition of the fecal microbiota of commercial mink between 2014 and 2015 was significantly different in community population (i.e., which bacterial taxa are present), based on the classic Jaccard index, and structure (i.e., which are present and their relative abundance), based on the Yue and Clayton index of dissimilarity (AMOVA and Parsimony *p*-values <0.001). These comparisons are depicted visually in Fig 2. Samples from 2014 were more even (Shannon’s evenness index) and diverse (Simpson’s index) than 2015 (*p* = 0.002 and 0.001, respectively) (Table 4).

**Table 4:** Alpha diversity indices (Chao 1: Richness; Shannon: Evenness; Inverse Simpson’s: Diversity) observed in the feces of commercial mink by year (n = 332), season (n = 117), and life stage (n = 332). Samples were obtained from 43, 46, and 39 farms in 2014, 2015, and 2016, respectively.

Parameters	Richness (Mean ± STD)	Evenness (Mean ± STD)	Diversity (Mean ± STD)
Year			
2014	192 ± 78	0.64 ± 0.11	14.6 ± 7.5
2015	197 ± 122	0.59 ± 0.15	12.2 ± 7.5
Life stage			
Adult females	183 ± 94	0.61 ± 0.12	13.0 ± 7.4
Weaned kits	201 ± 106	0.62 ± 0.14	13.7 ± 7.8
Season			
Summer	186 ± 115	0.59 ± 0.15	12.2 ± 7.6
Winter	157 ± 59	0.58 ± 0.15	13.1 ± 7.2

Comparisons of the relative abundance between 2014 and 2015 showed that 79 taxa were significantly different (n = 332), the greatest number of any of the comparisons. The median percent relative abundance, overall relative abundance cut-point for inclusion, and FDR *p*-values for the differences in years are summarized in Table 5. LEfSE analysis identified 109 OTUs as differentially enriched (LDA > 3; *p*<0.05).

**Table 5:** Relative abundance and false discovery rate *p*-values by year, for which at least 1 significant difference was identified between years from the fecal microbiota of mink, at each taxonomic level (n = 332). Samples were obtained from 43 and 46 farms in 2014 and 2015, respectively.

Taxonomic Level (cutoff)	Taxon	2014 Median% (Min-Max)	2015 Median% (Min-Max)	FDR *p*-value
Phylum (>0.1%)	Firmicutes	62.1 (11.9–97.2)	54.5 (8.9–98.8)	0.078
	Proteobacteria	33.1 (1.3–87.6)	31.4 (0.5–89.9)	0.722
	Bacteroidetes	0.2 (<0.1–7.8)	0.5 (<0.1–47.7)	<0.001
Class (>0.5%)	Bacilli	37.8 (6.2–91.4)	46.6 (6.0–97.2)	0.010
	Clostridia	17.1 (0.9–72.6)	5.4 (0.1–50.6)	0.001
	Erysipelotrichia	0.8 (<0.1–9.5)	0.4 (<0.1–6.8)	0.001
Order (>1%)	Xanthomonadales	14.1 (0.2–79.6)	4.8 (<0.1–84.2)	<0.001
	Clostridiales	17.0 (0.9–72.6)	5.2 (0.1–50.5)	<0.001
	Bacillales	4.6 (0.1–36.5)	7.7 (0.5–87.7)	<0.001
Family (>1.5%)	Xanthomonadaceae	14.1 (0.2–79.6)	4.8 (0.2–84.2)	<0.001
	Enterococcaceae	4.4 (0.2–22.2)	7.6 (0.4–25.1)	<0.001
	Peptostreptococcaceae	3.9 (<0.1–41.3)	1.3 (<0.1–28.9)	<0.001
	Carnobacteriaceae	4.3 (<0.1–35.7)	1.4 (<0.1–27.1)	<0.001
	Planococcaceae	1.0 (<0.1–20.7)	2.1 (0.1–37.5)	<0.001
	Clostridiales_Incertae_Sedis_XI	3.5 (<0.1–37.1)	0.4 (<0.1–18.4)	<0.001
Genus (>2%)	*Ignatzschineria*	15.4 (0.3–79.6)	7.2 (0–74.4)	<0.001
	*Enterococcus*	2.0 (<0.1–16.7)	4.8 (0.2–21.9)	<0.001
	*Escherichia*_*Shigella*	0.8 (0–44.3)	1.3 (<0.1–77.4)	0.003
	*Atopostipes*	4.2 (<0.1–35.1)	0.6 (0–19.0)	<0.001
	*Tissierella*	2.7 (0–22.9)	0.3 (0–14.9)	<0.001

### Mink fecal microbiota: Adult females 2014 vs 2015 and weaned kits 2014 vs 2015

Fecal microbiota of females from 2014 and 2015 were significantly different in population and structure, as was the fecal microbiota of kits from 2014 to 2015 (AMOVA and parsimony tests) (Table 6). These comparisons of population and structure by year and life stage are depicted visually by principal coordinate analyses (Fig 2) and dendrograms (S2 Fig). The microbiota of samples from adult females in 2014 were more even, but less diverse than the microbiota of samples from adult females in 2015 (*p* = 0.001 and <0.001, respectively), whereas there were no differences in alpha diversity parameters between samples from kits in 2014 compared to 2015. When comparing relative abundance, there were 64 taxa identified as significantly different between females in 2014 and 2015, while 59 taxa were identified as significantly different between kits in 2014 and 2015.

**Table 6:** Summary of differences in taxa, LEfSE analysis, and beta diversity indices between all comparison groups of the fecal microbiota of mink.

Parameters compared	Relative Abundance (# different taxa)	LEfSe (# differentially abundant OTUs)	Population (Jaccard Index *p*-values)		Structure (Yue & Clayton Index *p*-values)	
			AMOVA	Parsimony	AMOVA	Parsimony
Year						
2014–2015	79	109	<0.001	<0.001	<0.001	<0.001
Life stage						
F–K	30	40	0.008	0.430	<0.001	0.168
Life stage by year						
F14 –F15	64	NE	<0.001	0.001	<0.001	0.001
F14 –K14	0	NE	0.031	0.643	0.097	0.824
F15 –K15	0	NE	0.181	0.334	0.029	0.238
K14 –K15	59	NE	<0.001	0.001	<0.001	0.001
Season						
F15 –F16	78	88	<0.001	0.001	<0.001	0.001

AMOVA, analysis of molecular variance; NE, not examined

F = adult females, K = weaned kits, followed by the year (summers 2014 and 2015, winter 2016) from which the sample was taken

### Mink fecal microbiota: Adult females vs weaned kits

Fecal microbiota of samples from females and kits from 2015 were not significantly different in population (AMOVA and parsimony test) or structure (parsimony test). Similarly, females and kits from 2014 were similar in structure (AMOVA and parsimony tests) and in population (parsimony test) (Table 6). When 2014 and 2015 were combined, the community population (Jaccard Index) and structure (Yue and Clayton Index) of the fecal microbiota from adult females and weaned kits were significantly different by AMOVA, but not parsimony (Table 6). Comparison of population and structure by year and life stage are depicted visually by principal coordinate analyses (Fig 2) and dendrograms (S2 Fig). There were no differences in alpha diversity measurements between adult females and weaned kits.

When assessing relative abundance, the genus *Ignatzschineria* (phylum Proteobacteria) was significantly more abundant in adult females than weaned kits (median = 11.4% and 6.2%, respectively; *p* = 0.004), as was its corresponding family, order and phylum (S1 Table). The genus *Atopostipes* was significantly more abundant in adult females than weaned kits (median = 1.9% and 1.0%, respectively; *p* = 0.027). The genus *Enterococcus* (phylum Firmicutes) was significantly less abundant in adult females than weaned kits (median = 4.7% and 6.7%, respectively; *p* = 0.036), as was its corresponding family. No taxonomic differences in relative abundance were identified between adult females and weaned kits from the same year (Table 6). Additional taxa differences by life stage are available in S1 Table.

**Fig 3:**
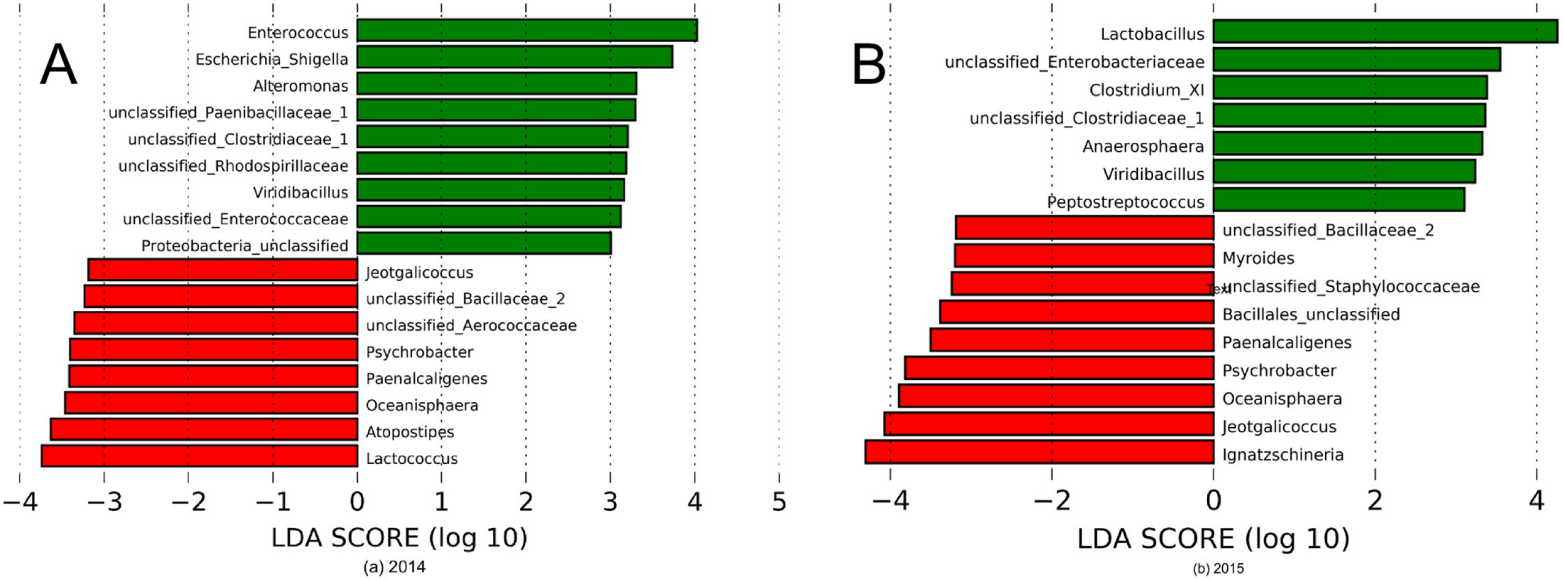
Linear discriminant analysis effect size (LEfSe) indicating differentially enriched fecal bacterial communities (with LDA score >3) at the genus level between adult females (red, n = 164) and weaned kits (green, n = 168) in summer 2014 (a) and summer 2015 (b) (n = 332). Samples were obtained from 43 and 46 farms in 2014 and 2015, respectively.

LEfSE analysis identified 40 OTUs as differentially abundant between adult females and weaned kits (*p*<0.05); those with LDA scores >3 are depicted in Fig 3. All except one of the OTUs enriched in weaned kits were Firmicutes, of which only the orders Lactobacillales and Clostridiales were represented, with the exception of one OTU from the order Tissierellales.

### Mink fecal microbiota: Adult females summer 2015 vs winter 2016

The composition of the fecal microbiota of commercial mink was significantly different in community population and structure by season (AMOVA and Parsimony *p*-values <0.001) (Table 6). There were no differences in alpha diversity measurements when comparing season.

**Fig 4:**
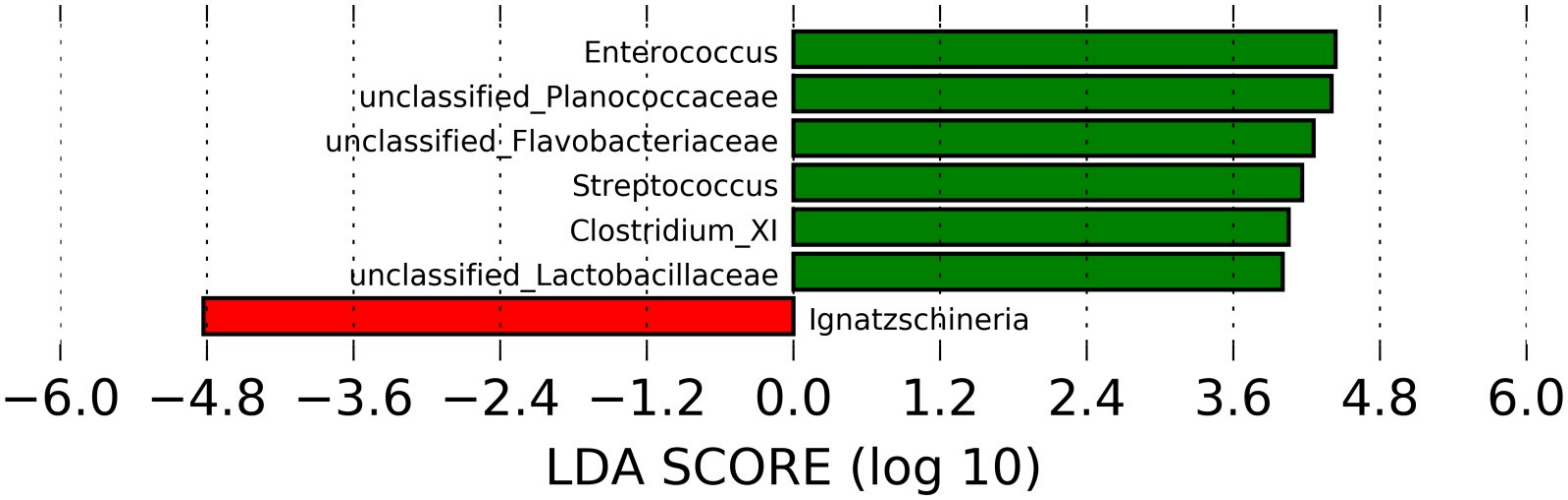
Linear discriminant analysis effect size (LEfSe) indicating differentially enriched fecal bacterial communities (with LDA score >3) at the genus level of adult females by season (n = 117). Green = winter (n = 34), red = summer (n = 83). Samples were obtained from 46 and 39 farms in 2015 and 2016, respectively.

The relative abundance of all three of the predominant phyla were significantly different, with Firmicutes and Bacteroidetes significantly higher in winter (*p* = <0.001 and 0.025, respectively), and Proteobacteria higher in summer (*p* = <0.001). The genus *Enterococcus* (Firmicutes) had a higher relative abundance in samples from winter than summer (*p* = 0.013), as did its corresponding order (Lactobacillales, *p* = 0.044) and class (Bacilli, *p* = 0.020). During summer, samples were found to have a significantly higher relative abundance of the genus *Ignatzschineria* (*p* = <0.001), as well as its corresponding family, order, and class. The order Clostridiales and class Clostridia (Firmicutes) had a higher relative abundance in samples from winter than in samples from summer (*p* = 0.007 and 0.012, respectively) (S2 Table). LEfSE analysis identified 88 OTUs that were differentially abundant between adult females in summer and winter (*p*<0.05); those with LDA scores >3 are depicted in Fig 4.

### Mink fecal microbiota: Individual farms 2014 vs 2015

A total of 30 farms had complete data for 2014 and 2015 (i.e., two pooled adult female samples and two pooled weaned kit samples from both years, for a total of eight pooled samples from each farm) and each of these farms was individually compared between 2014 and 2015. The 9 and 15 predominant phyla and genera, respectively, were compared by farm. Of the 30 farms, 11 (37%) had zero and 6 (20%) had only one phylum, out of those examined, which were significantly different between 2014 and 2015. When evaluating only those phyla for which the overall relative abundance was >1.0% (Firmicutes, Proteobacteria, and Bacteroidetes), 17 of 30 farms (57%) had no significant differences and 11 (37%) had only one phylum that was significantly different between 2014 and 2015. When only the two predominant phyla are considered (i.e., Firmicutes and Proteobacteria), together accounting for >95% of the total sequences, only 5 farms had one or both phyla that were significantly different between 2014 and 2015. Overall, 83% (223/270) of the comparisons made were similar between years, by farm, and by phylum. Similarly, 80% of the genera had similar relative abundance by farm from 2014 to 2015.

## Discussion

The predominant bacterial phylum in the feces of commercial mink overall, regardless of year, life stage, or season, was Firmicutes. However, Firmicutes is followed by a much larger proportion of Proteobacteria than has been previously reported in healthy dogs, cats, and other carnivores (34% vs <15%) [7–9, 28–33]. This is consistent with the high relative abundance of both Firmicutes and Proteobacteria found in mink feces by Bahl *et al*., but unlike the work of Zhao *et al*., in which a higher overall relative abundance of Proteobacteria was seen in mink feces, although the latter used a different methodology (amplification of the V3 region), which may explain, at least in part, the lack of agreement between studies [15,34]. As seen in other mammals, when the microbiota is compared between adults and young weaned animals [35–39], there were few significant differences identified between adult female mink and weaned kits.

At the order level, organisms from Lactobacillales comprise more than one-third of the total bacterial sequences identified from mink feces, more than twice the next most abundant order, Xanthomonadales. Lactobacillales, to which the genus *Lactobacillus* belongs, consists primarily of a group of aerotolerant anaerobic bacteria that produce lactic acid as the end-product of carbohydrate fermentation. This is an unexpected finding, as previous studies characterizing the fecal microbiota of cats and pigs found a much lower relative abundance and total count of lactic acid bacteria, respectively [35–37] as well as a significant, age-related decrease in these bacteria. The results of the latter study are in contrast to Frese *et al*, who found a significant increase in the relative abundance of lactic acid bacteria from nursing to weaning [38]. In our study, although weaned kits had a higher overall proportion of Lactobacillales than adult females, this difference was not significant. Interestingly, while the relative abundance of Lactobacillales was not significantly different between the groups, specific OTUs of the genera *Lactococcus* and *Lactobacillus* were both significantly enriched in weaned mink kits, and the former had a significantly higher relative abundance in weaned kits than adult females. This suggests that while the composition of the microbiota in the weaned population that the samples came from was likely close to maturity, there was persistence of certain genera that are typically associated with preweaned animals [39–41].

In this study, a significantly higher proportion of the class Clostridia was identified in the feces of mink in 2014 compared to 2015 (17% vs 5%, respectively). In 2014, there was a North American-wide shortage of the *Clostridium botulinum* toxoid vaccine, which is typically given to mink kits at approximately 10 weeks of age as well as adult females. It is unclear, however, the mechanism that would have caused this increase, as vaccination against the toxoid is unlikely to impact the presence of multiple clostridial species within the gut. It is unknown if there was a corresponding increase in morbidity or mortality due to infection with *Clostridium* spp. in 2014. In the future, correlating changes in standard preventative care practices with both the clinical outcomes and the effects on the composition of the fecal microbiome would be useful.

An unexpectedly high proportion of organisms were identified from the genus *Ignatzschineria* and the only genus of the phylum Proteobacteria that was enriched in weaned kits was *Wohlfahrtiimonas*, which is closely related to *Ignatzschineria* [42]. Several species from these genera have been associated with human maggot infestation and associated bacteremia [43,44]. *Ignatzschineria* was first identified from the gut of an obligate parasitic fly larvae, *Wohlfahrtia magnifica* [45]. This suggests that the finding of this genus in the current study represents contamination of fecal samples from flies. Further supporting this is the significant decrease in the overall relative abundance of this genus from summer to winter, when most environmental insects are eliminated. Moreover, Bahl *et al* found that the fecal microbiota of mink does not cluster with the microbiota of the host’s feed source [15], providing additional evidence that the high relative abundance of *Ignatzschineria* was likely the result of contamination of the feces (rather than the food source) with flies. However, we cannot definitively rule out that these organisms are true inhabitants of the mink gut, potentially from contaminated feed.

The impact of year appeared to be more significant than life stage or season, and animals from the same year were similar in all regards. The mink fecal microbiota can likely be altered by a range of dietary, environmental, management, and treatment factors. It is unsurprising that the largest number of differences were identified between 2014 and 2015, as these years would have had the fewest number of the same individuals contributing to the samples collected, particularly when compared to the adult females in summer of 2015 and winter of 2016 in which there were likely some of the same animals being sampled. This is consistent with data from healthy adult humans, in which the fecal microbiota of an individual is more similar to itself over time, than to another’s fecal microbiota [46–49]. Additionally, mink diets are heavily reliant on meat and fish by-product commodity prices as well as the availability of other protein sources, such as cracked eggs, livestock offal, etc., which may not be consistent from year-to-year or even season to season. The differences observed in fecal microbiota composition over the three years studied may be a reflection of widely variable diets or other management practices not examined.

At the farm-level, the predominant phyla and genera were found to be similar from 2014 to 2015. This is consistent with data from humans; individuals and family members (who have a shared environment) have a more similar fecal and oral microbiota than do unrelated individuals [46,50,51]. The implications of this is that there is more value in sampling from a larger number of environments (e.g. more farms) than a greater number of individuals from the same environment. It has additionally been shown that the skin but not the fecal microbiota of owners and their cohabitating pets were more similar than non-cohabitating pets [51]. We have previously determined that hygiene and biosafety are generally poor on many Canadian mink farms [52]. It would be interesting to determine if there are corresponding findings in the skin or fecal microbiota of mink farm workers.

The mink gut is very short with a highly acidic stomach, which may make them more resistant to bacteria in decomposing meat and other foods [10,53]. In the present study, a much higher proportion of Proteobacteria was identified than has previously been seen in other carnivores, regardless of year, life stage, or season. Bahl *et al*. found a similar high relative abundance of Proteobacteria, with Firmicutes still having the overall highest relative abundance [15]. Our findings are in contrast to Zhao *et al*. in which Proteobacteria were of highest overall relative abundance and Firmicutes were second highest [34]. However, the latter study used the V3 region of the 16S rRNA gene (*vs* the V4 region used in the current study), which may result in less stringent fragment detection, so their findings are likely not directly comparable and may account for these differences. In humans, increases in the relative abundance of Proteobacteria have been associated with metabolic disorders, enteric inflammation, and dysbiosis [54–57]. Additionally, metagenomic gene families with very high variance in abundance across hosts were almost entirely specific to the phylum Proteobacteria, whereas those invariable gene families (i.e. low variance across hosts) were generally specific to Firmicutes and Bacteroidetes, indicating that in the human gut the abundance of Proteobacteria may influence functional variability [58]. Feces selected for this study came from apparently healthy animals, yet showed a very high relative abundance of Proteobacteria. In both humans and calves, recent antibiotic use has been associated with increased relative abundance of Proteobacteria [59, 60]. Day to day antimicrobial use was not reported at feces collection and it is difficult to assess if this may have contributed to the high levels of Protebacteria.

A shortcoming of this study is related to how fecal samples were collected. Some samples may have been fresher than others, collected at different times of day or at different times in relation to husbandry activities. Thus, there may have been differences in environmental exposure and potential contamination. Additionally, samples were not aliquoted immediately at collection, resulting in an additional freeze-thaw cycle. Song *et al* found that the fecal microbiome can shift over 8-weeks when subjected to freeze-thaw temperature fluctuations; however, the resulting effect size was generally less than the variation between individuals [61]. Regardless, the use of a preservative was found to reliably stabilize the microbiome and should be considered for future projects where temperature fluctuations or prolonged storage are needed [61]. Finally, it is unknown if the mink fecal microbiota is representative of the microbiota of other sites in the gastrointestinal tract. Pang *et al* determined that the microbiota of the cecum does not cluster with the fecal microbiota in mice [62]. Similarly, distinct but overlapping microbial communities were identified along the gastrointestinal tract of dogs and cats, and, in the latter, the microbiota clustered by individual, rather than by body site [33,63]. Thus, although study of the fecal microbiota offers a noninvasive technique additional studies looking at other sites within the gastrointestinal tract of mink are needed to determine whether gut microbial communities are similar.

In conclusion, we have shown that individual sampling year has more of an impact on the mink fecal microbiota than life stage or season, despite the substantial change in farmed mink diet from summer to winter. Furthermore, by characterizing the mink fecal microbiota, we have provided a baseline for the future study of how the normal composition is altered in disease states and, potentially, how the microbiota can be used to optimize production of farmed mink. The use of probiotics has been shown, for example, to increase overall productivity when given to growing broiler chickens and also result in increased daily weight gain in weaning piglets [20,21]. As pre-weaning mortality represents a significant loss to mink farmers in Canada, and the odds of dying decrease with increasing body weight during the preweaning period, promotion of weight gain through the use of probiotics could represent a means to increase production and animal welfare overall [52]. Better food handling practices and manure and pest control programs may also improve the quality of food fed as well as the overall environment, enhancing animal growth and well-being.

## Supporting information

S1 Fig Relative abundances of 20 most predominant bacterial genera present in the feces of Canadian commercial mink, by farm (n = 49). (TIF)

S2 Fig Dendrogram representing a) the community population and (b) structure of the fecal microbiota of commercial mink, based on the Jaccard Index and Yue and Clayton Index, respectively (red = 2014, green = 2015, 2016 = blue). (TIF)

S1 Table Relative abundance and false discovery rate (FDR) *p*-values for significantly different taxa (*p*<0.05) by life stage in mink fecal microbiota (n = 332; 2014 and 2015 only). (DOCX)

S2 Table Relative abundance and false discovery rate (FDR) *p*-values for significantly different taxa (*p*<0.05) by season in the fecal microbiota of mink (n = 117; adult females 2015 and 2016 only). (DOCX)
