# Supplementary figures and images for: Fecal bacterial microbiota of Canadian commercial mink (Neovison vison): Yearly, life stage, and seasonal comparisons
Source: PLoS One. 2018 Nov 12;13(11):e0207111. doi: 10.1371/journal.pone.0207111 (PMC6231641; doi:10.1371/journal.pone.0207111)

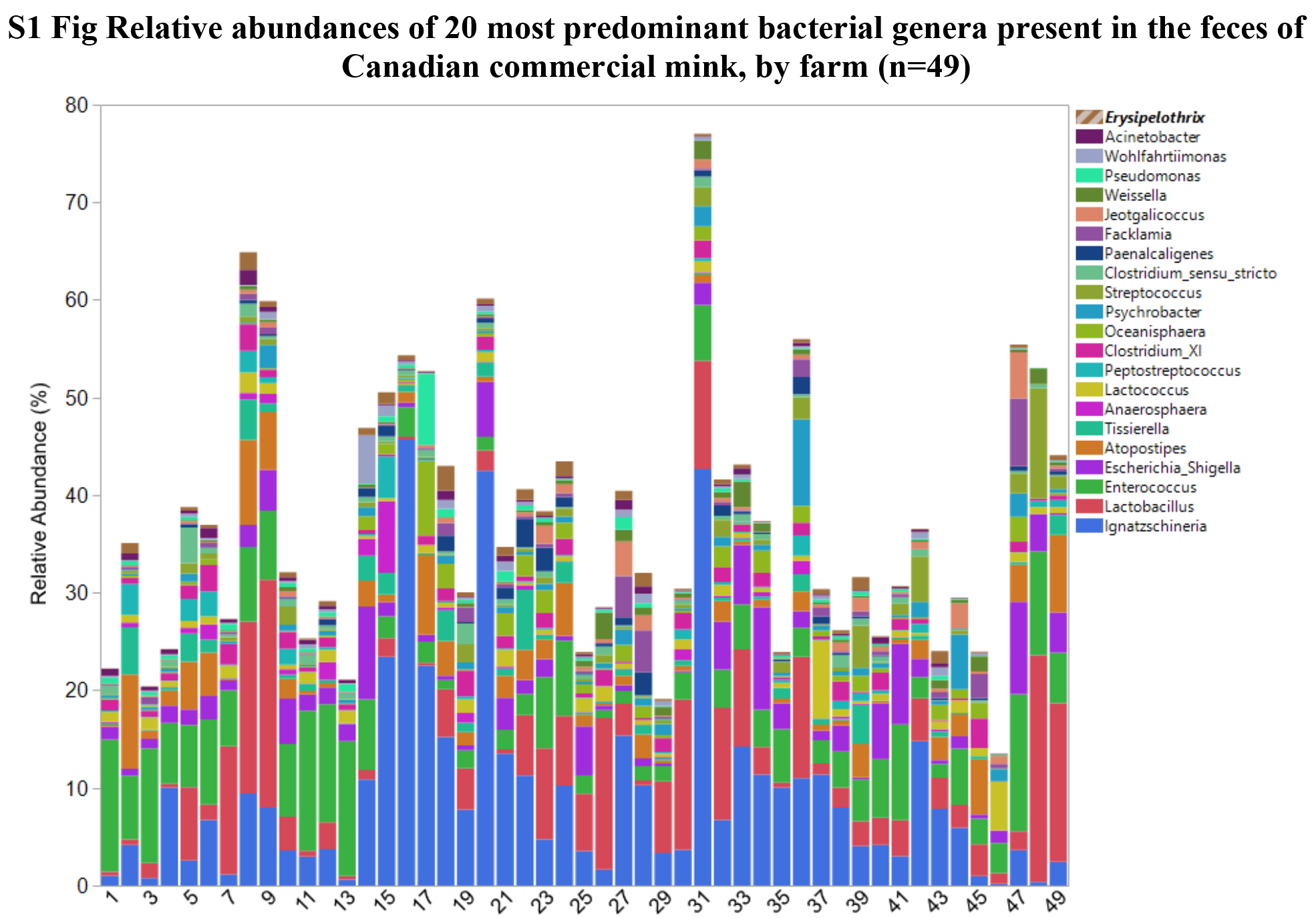

Supplement: S1 Fig — (TIF) [file pone.0207111.s001.tif]

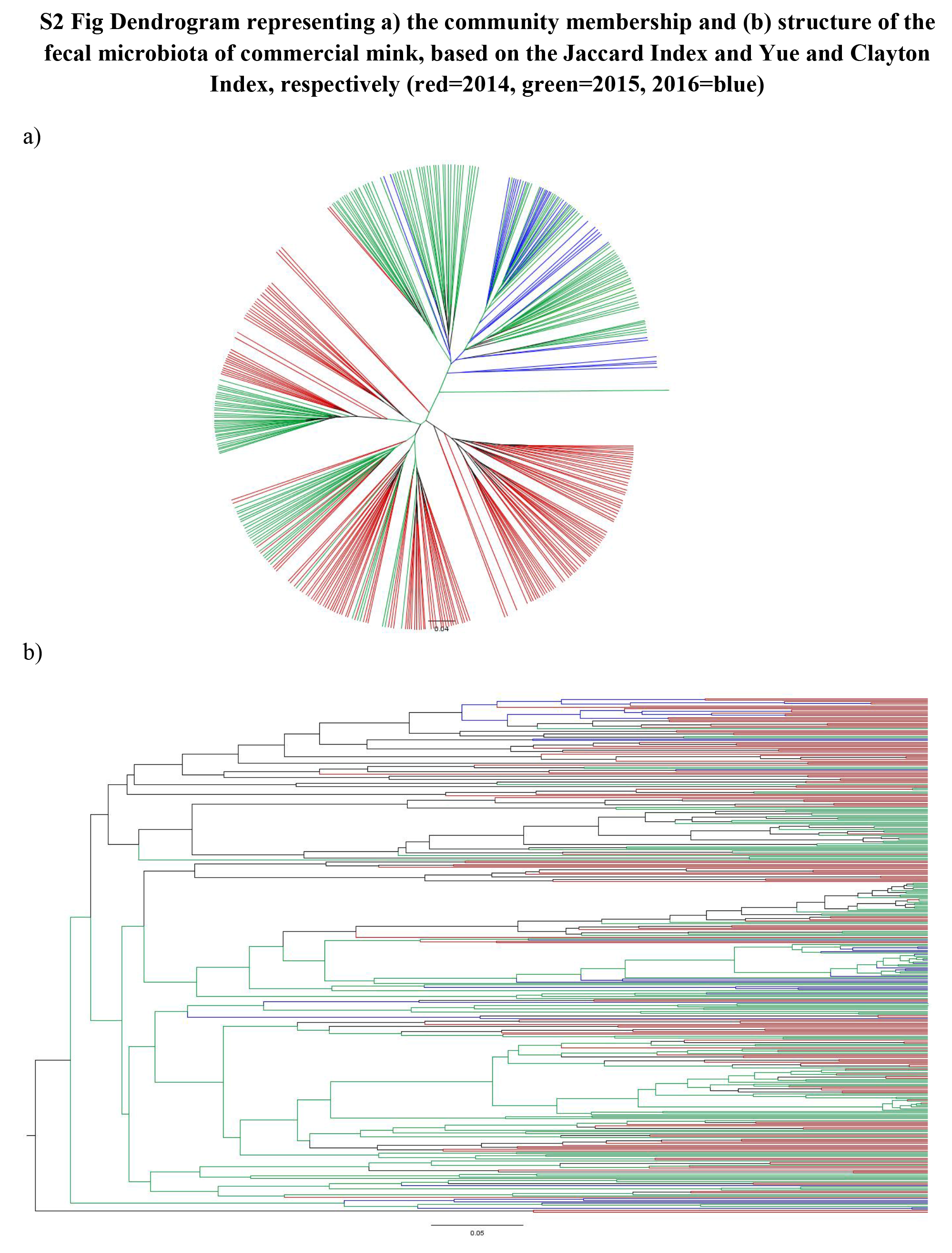

Supplement: S2 Fig — (TIF) [file pone.0207111.s002.tif]
