# Supplementary material for: Fecal bacterial microbiota of Canadian commercial mink (Neovison vison): Yearly, life stage, and seasonal comparisons
Source: PLoS One. 2018 Nov 12;13(11):e0207111. doi: 10.1371/journal.pone.0207111 (PMC6231641; doi:10.1371/journal.pone.0207111)
Supplement: S1 Table — (DOCX) [file pone.0207111.s003.docx]

**S1 Table. Relative abundance and false discovery rate (FDR) *p-*values for significantly different taxa (*p*<0.05) by life stage in mink fecal microbiota (n=332; 2014 and 2015 only).**

| **Taxonomic Level (overall cutoff)** | **Taxon** | **Adult Female**  **Median %**  **(Min-Max)** | **Weaned Kit**  **Median %**  **(Min-Max)** | **FDR**  ***p*-value** |
| --- | --- | --- | --- | --- |
| Phylum  (>0.1%) | Proteobacteria | 35.1  (1.1 – 87.6) | 29.5  (0.5 – 89.9) | 0.0440 |
|  | Fusobacteria | <0.1  (<0.1 – 4.3) | <0.1  (<0.1 – 14.1) | 0.0440 |
| Class  (>0.1%) | Gammaproteobacteria | 29.7  (1.1 – 87.6) | 22.4  (0.5 – 88.4) | 0.0301 |
|  | Betaproteobacteria | 0.6  (<0.1 – 30.1) | 0.2  (<0.1 – 15.2) | 0.0182 |
|  | Fusobacteria | <0.1  (<0.1 – 4.3) | <0.1  (<0.1 – 14.1) | 0.0411 |
| Order  (>0.1%) | Xanthomonadales | 11.8  (<0.1 – 79.6) | 6.9  (<0.1 – 84.2) | 0.0331 |
|  | Pseudomonadales | 2.9  (<0.1 – 56.3) | 2.0  (<0.1 – 73.4) | 0.0063 |
|  | Aeromonadales | 0.8  (<0.1 – 43.9) | 0.2  (<0.1 – 29.7) | 0.0061 |
|  | Burkholderiales | 0.5  (<0.1 – 30.1) | 0.2  (<0.1 – 15.1) | 0.0061 |
|  | Fusobacteriales | <0.1  (<0.1 – 4.3) | <0.1  (<0.1 – 14.1) | 0.0061 |
| Family  (>0.2%) | Xanthomonadaceae | 11.8  (0 – 79.6) | 6.9  (<0.1 – 84.2) | 0.0360 |
|  | Enterococcaceae | 4.7  (0.2 – 25.1) | 6.7  (0.3 – 23.4) | 0.0360 |
|  | Carnobacteriaceae | 3.4  (<0.1 – 35.7) | 2.2  (<0.1 – 33.9) | 0.0273 |
|  | Streptococcaceae | 1.3  (<0.1 – 43.5) | 1.6  (<0.1 – 48.0) | 0.0473 |
|  | Moraxellaceae | 1.3  (<0.1 – 39.4) | 0.8  (<0.1 – 73.2) | 0.0473 |
|  | Staphylococcaceae | 1.0  (<0.1 – 85.6) | 0.4  (<0.1 – 34.0) | 0.0062 |
|  | Incertae_Sedis_XI | 0.1  (<0.1 – 39.2) | 0.3  (<0.1 – 37.1) | 0.0400 |
|  | Aerococcaceae | 1.0  (<0.1 – 35.0) | 0.5  (<0.1 – 17.9) | 0.0360 |
|  | Aeromonadaceae | 0.8  (<0.1 – 43.9) | 0.2  (<0.1 –29.7) | 0.0016 |
|  | Alcaligenaceae | 0.5  (<0.1 – 3<0.1) | 0.1  (<0.1 – 15.1) | 0.0016 |
|  | Bacillaceae_2 | 0.2  (<0.1 – 13.1) | 0.1  (<0.1 – 11.8) | 0.0093 |
| Genus  (>1.1%) | Ignatzschineria | 11.4  (0 – 79.6) | 6.2  (0.2 – 79.0) | 0.0234 |
|  | Enterococcus | 3.0  (<0.1 – 21.9) | 5.1  (0.1 – 19.8) | 0.0267 |
|  | Atopostipes | 1.9  (0 – 35.1) | 1.0  (0 – 33.4) | 0.0267 |
|  | Anaerosphaera | 0.1  (0 – 38.5) | 0.2  (0 – 27.8) | 0.0267 |
|  | Lactococcus | 0.6  (<0.1 – 40.5) | 1.0  (0 – 44.1) | 0.0234 |
|  | Oceanisphaera | 0.7  (0 – 44.0) | 0.1  (0 – 3<0.1) | 0.0015 |
|  | Psychrobacter | 0.5  (0 – 39.4) | 0.3  (0 – 73.2) | 0.0240 |
|  | Paenalcaligene | 0.5  (0 – 27.5) | 0.1  (0 – 14.8) | 0.0020 |
|  | Jeotgalicoccus | 0.4  (0 – 53.0) | 0.1  (0 – 11.6) | 0.0015 |
