# Supplementary material for: Fecal bacterial microbiota of Canadian commercial mink (Neovison vison): Yearly, life stage, and seasonal comparisons
Source: PLoS One. 2018 Nov 12;13(11):e0207111. doi: 10.1371/journal.pone.0207111 (PMC6231641; doi:10.1371/journal.pone.0207111)
Supplement: S2 Table — (DOCX) [file pone.0207111.s004.docx]

**S2 Table. Relative abundance and false discovery rate (FDR) *p-*values for significantly different taxa (*p*<0.05) by season in the fecal microbiota of mink (n=117; adult females 2015 and 2016 only).**

| **Taxonomic Level (overall cutoff)** | **Taxon** | **Summer**  **Median %**  **(Min-Max)** | **Winter**  **Median %**  **(Min-Max)** | **FDR**  ***p*-value** |
| --- | --- | --- | --- | --- |
| Phylum  (>0.1%) | Firmicutes | 53.3  (10.9 – 98.8) | 65.4  (11.5 – 99.0) | 0.001 |
|  | Proteobacteria | 35.1  (1.1 – 86.1) | 25.6  (0.6 – 88.2) | 0.001 |
|  | Bacteroidetes | 0.7  (0 – 47.7) | 5.3  (0 – 22.8) | 0.031 |
|  | Acidobacteria | 0  (0 – 3.2) | 0  (0 – 0) | 0.009 |
|  | Fusobacteria | 0  (0 – 4.3) | 0.1  (0 – 0.8) | 0.016 |
|  | Verrucomicrobia | 0  (0 – 2.0) | 0  (0 – 0) | 0.001 |
|  | Chlamydiae | 0  (0 – 0.5) | 0  (0 – 0.1) | 0.014 |
| Class  (>0.1%) | Bacilli | 40.4  (6.4 – 97.2) | 51.2  (10.8 – 96.1) | 0.020 |
|  | Gammaproteobacteria | 29.7  (1.1 – 87.6) | 22.8  (0.5 – 88.1) | 0.004 |
|  | Clostridia | 4.9  (0.1 – 38.1) | 7.0  (0.1 –55.6) | 0.012 |
|  | Alphaproteobacteria | 0.7  (0 – 10.6) | 0.4  (0 –1.4) | 0.028 |
|  | Flavobacteria | 0.4  (0 – 43.2) | 4.1  (0 –16 6.) | 0.023 |
| Order  (>0.1%) | Lactobacillales | 22.3  (4.3 – 91.7) | 37.5  (1.5 – 71.4) | 0.044 |
|  | Xanthomonadales | 8.0  (0 – 74.5) | 2.0  (<0.1 – 5.3) | <0.001 |
|  | Clostridiales | 4.9  (0.1 – 38.1) | 6.7  (0.1 – 55.2) | 0.007 |
|  | Bacillales | 5.9  (0.1 – 87.7) | 11.0  (2.6 – 93.1) | 0.012 |
|  | Flavobacteriales | 0.4  (0 – 43.2) | 4.1  (<0.1 – 16.6) | 0.017 |
| Family  (>0.2%) | Xanthomonadaceae | 11.8  (<0.1 – 79.6) | 2.0  (0 – 5.3) | <0.001 |
|  | Peptostreptococcaceae | 1.2  (0 – 28.9) | 2.5  (0.1 – 27.8) | <0.001 |
|  | Planococcaceae | 1.9  (0.4 – 33.5) | 5.2  (0.9 – 85.6) | <0.001 |
|  | Streptococcaceae | 1.4  (0 – 25) | 3.1  (0 – 31.1) | 0.005 |
|  | Pseudomonadaceae | 1.0  (0 – 9.6) | 1.5  (0.1 – 43.4) | 0.031 |
|  | Clostridiaceae_1 | 0.5  (0 – 12.3) | 2.6  (<0.1 – 25.4) | <0.001 |
|  | Aerococcaceae | 0.5  (0 – 35.0) | 0.1  (0.1 – 0.4) | <0.001 |
| Genus  (>1.1%) | *Ignatzschineria* | 7.2  (0 – 74.4) | 1.9  (<0.1 – 4.9) | <0.001 |
|  | *Enterococcus* | 4.8  (0.2 – 21.9) | 7.3  (0.3 – 53.3) | 0.0133 |
|  | *Clostridium_*XI | 0.4  (0 – 19.7) | 1.7  (0 – 27.0) | <0.001 |
|  | *Streptococcus* | 0.2  (0 – 24.7) | 2.6  (0 – 30.7) | <0.001 |
|  | *Facklamia* | 0.1  (0 – 30.7) | <0.1  (0 – 0.3) | <0.001 |
|  | *Pseudomonas* | 0.3  (0 – 1.9) | 0.4  (<0.1 – 11.7) | 0.038 |
|  | *Wohlfahrtiimona* | 0.1  (0 – 20.0) | <0.1  (0 – 0.4) | <0.001 |
|  | *Acinetobacter* | 0.1  (0 – 25.3) | <0.1  (0 – 2.5) | <0.001 |
